# Changes in handwashing knowledge, attitudes, and practices among primary schoolchildren in Ulaanbaatar, Mongolia during the COVID-19 pandemic

**DOI:** 10.3389/fpubh.2025.1570178

**Published:** 2025-06-12

**Authors:** Munguntuul Enkhbat, Ganchimeg Togoobaatar, Oyunchimeg Erdenee, Katsumata Asako Takekuma

**Affiliations:** ^1^Department of Public Health Nursing, Mongolian National University of Medical Sciences, Ulaanbaatar, Mongolia; ^2^Department of Global Health Nursing, Institute of Medicine, University of Tsukuba, Tsukuba, Japan; ^3^Department of Physical Education, Mongolian National Institute of Physical Education, Ulaanbaatar, Mongolia; ^4^Department of Health Research, Graduate School, Mongolian National University of Medical Sciences, Ulaanbaatar, Mongolia; ^5^Department of Global Health Nursing & Nursing Leadership, University of Shizuoka, Shizuoka, Japan

**Keywords:** handwashing, schoolchildern, knowledge, attitude, and practice (KAP), COVID-19, Mongolia

## Abstract

**Introduction:**

The coronavirus disease 2019 (COVID-19) pandemic has highlighted the critical importance of handwashing as a preventive measure against the spread of SARS- CoV-2. This study aimed to assess changes in handwashing knowledge, attitudes, and practices (KAP) among primary schoolchildren in Ulaanbaatar, Mongolia, before and during the pandemic.

**Methods:**

A longitudinal study was conducted with 399 children aged 8–11 years, with data collected in December 2019 (pre-pandemic) and January 2021 (during the pandemic). A self-reported questionnaire was used to assess the children’s handwashing KAP, focusing on four critical moments for handwashing: after using the toilet, before eating, after touching visibly dirty or unhygienic things, and after coming home.

**Results:**

The results showed a significant increase in handwashing knowledge and attitudes during the pandemic, with the average knowledge score rising from 9.8 to 12.3 and the percentage of correct responses about critical handwashing moments increasing from 23.0 to 80.4%. Handwashing practices also improved, with an increased frequency of handwashing at critical moments, although practice before eating remained unchanged. Sociodemographic factors, such as sex, grade level, and parental education, were associated with handwashing behaviors. Girls were significantly more likely to engage in proper handwashing (AOR = 4.50, *p* < 0.01), while fourth-grade students showed higher odds of practicing proper handwashing than third-grade students (AOR = 5.27, *p* < 0.01). Fathers’ higher education and maternal self-employment were positively associated with proper handwashing, whereas fathers’ employment in public service was negatively associated. The overall KAP scores showed a significant increase during the pandemic, with a mean difference of 3.68 [95% CI = (4.06, 3.29), *p* < 0.01], indicating a notable improvement in handwashing behavior.

**Discussion:**

These findings highlight the importance of health education and interventions in shaping children’s hygiene behaviors during global health crises, with lasting implications for public health strategies, particularly in resource-limited settings.

## Introduction

In response to COVID-19, countries worldwide have implemented various public health measures, including personal hygiene actions such as handwashing, social distancing, and wearing masks (1). The majority of countries have implemented multiple infection control measures, such as personal actions (social distancing, washing or sanitizing hands, and wearing masks), case and contact identifications (testing and tracking of transmission), regulatory actions (restrictions and closure orders, etc.), and international border measures (2). Among the key preventive actions, hand hygiene plays a critical role in limiting the transmission of SARS-CoV-2. While most studies have focused on adult populations, children’s adherence to handwashing guidelines and their ability to practice hygiene behaviors are crucial, especially in school environments where close conduct is inevitable. Until an effective vaccine is available, behavioral interventions such as hand hygiene, mask-wearing, and social distancing are critical in controlling transmission (3, 4). Prior to the World Health Organization’s emergency use authorization of qualified COVID-19 vaccines and effective therapeutic drugs, options for young children were limited, making preventive measures such as handwashing and mask-wearing the most powerful tools for combating the virus (4).

Handwashing with soap is a proven and cost-effective intervention for reducing the transmission of both gastrointestinal and respiratory infections, making it especially vital during the COVID-19 pandemic (5, 6). For children, who are often more susceptible to infections, this measure is critical in preventing viral transmission. Despite this, studies suggest that children’s adherence to hygiene practices varies widely according to the context, education, and resources available.

Studies have also concluded that the prevalence of handwashing facilities is important during the novel COVID-19 pandemic, especially in low-income countries, along with handwashing with soap, wearing masks, and social distancing (7). Mongolia, located near China, implemented early measures such as border restrictions and strict quarantine protocol starting on 28 January 2020, to prevent the spread of COVID-19. The government also closed schools, promoted public health measures, and prevented local transmission for 8 months following the first imported case on 10 March 2020. The Mongolian government has implemented community-based public health interventions such as travel restrictions, closure of all educational institutions, health promotion, and disease prevention activities. Schools in Mongolia remained closed from January 2020 to September 2020, with restrictions reimposed from November 2020 due to local outbreaks. During this period, education shifted to distance learning and limited opportunities for children to practice hand hygiene in school settings (8). During the lockdown, the government conducted regular press conferences (92.6% of households had a television) to provide daily updates on the pandemic and call for preventive measures including handwashing, mask-wearing, and social distancing (9). In addition to television, the social network of an official website or page provides health education and promotion on a regular basis (10, 11). In Mongolia, early interventions such as school closures, border controls, health education campaigns the transmission of the virus, and the long-term effect on children’s health behavior, particularly hand hygiene, are less understood. With schools closed for extended periods, children had limited opportunities to practice hand hygiene in educational settings, making it crucial to evaluate how these disruptions affected their KAP regarding handwashing.

Many studies have explored the mechanisms, prognosis, treatments, and preventive actions of COVID-19 (2, 12, 13), and it has been studied more in older adults aged ≥ 65 years. There is limited evidence among young children, especially primary school-aged children. There are multiple prevention and infection control measures for the transmission of COVID-19; however, we aimed to explore the impact of COVID-19 on handwashing behavior among elementary school children. By comparing these behaviors before and during the pandemic, we seek to fill a critical gap in understanding the factors influencing children’s hygiene practices during the global health crisis. The initial aim of this study was to assess the effect of an interactive intervention on the study population; however, the planned schedule could not be implemented because of disruptions caused by the COVID-19 pandemic. Originally designed to evaluate handwashing knowledge, attitude, and practice (KAP) among this population, the study was unanticipated in the context of the pandemic, which brought heightened global attention to hygiene behavior, particularly among children. Our study was more concerned with the impact of handwashing behavior among schoolchildren before and during the COVID-19 pandemic. The KAP model is fundamental to understanding children’s engagement in health-promoting behaviors. In the context of COVID-19, KAP measures can identify gaps in children’s understanding, attitudes, and behavior related to hygiene, which are critical for preventing the spread of the virus.

## Materials and methods

A school-based longitudinal study was conducted to examine differences in handwashing KAP among primary school-aged children before (December 2019) and during (January 2021) the COVID-19 pandemic. The study followed the children who were enrolled in 2019, aged between 8 and 11 years.

### Study setting

The study was conducted in a public school in the Bayangol District of Ulaanbaatar, Mongolia. Bayangol is the largest of the nine districts, accounting for 15.6% of Ulaanbaatar’s population, with 18 public schools. The enrolled school primarily serves children from surrounding apartment complexes, with sociodemographic characteristics similar to those of other central districts of Ulaanbaatar, where the majority of households have access to central water sewage systems.

### Participants

**Figure 1:**
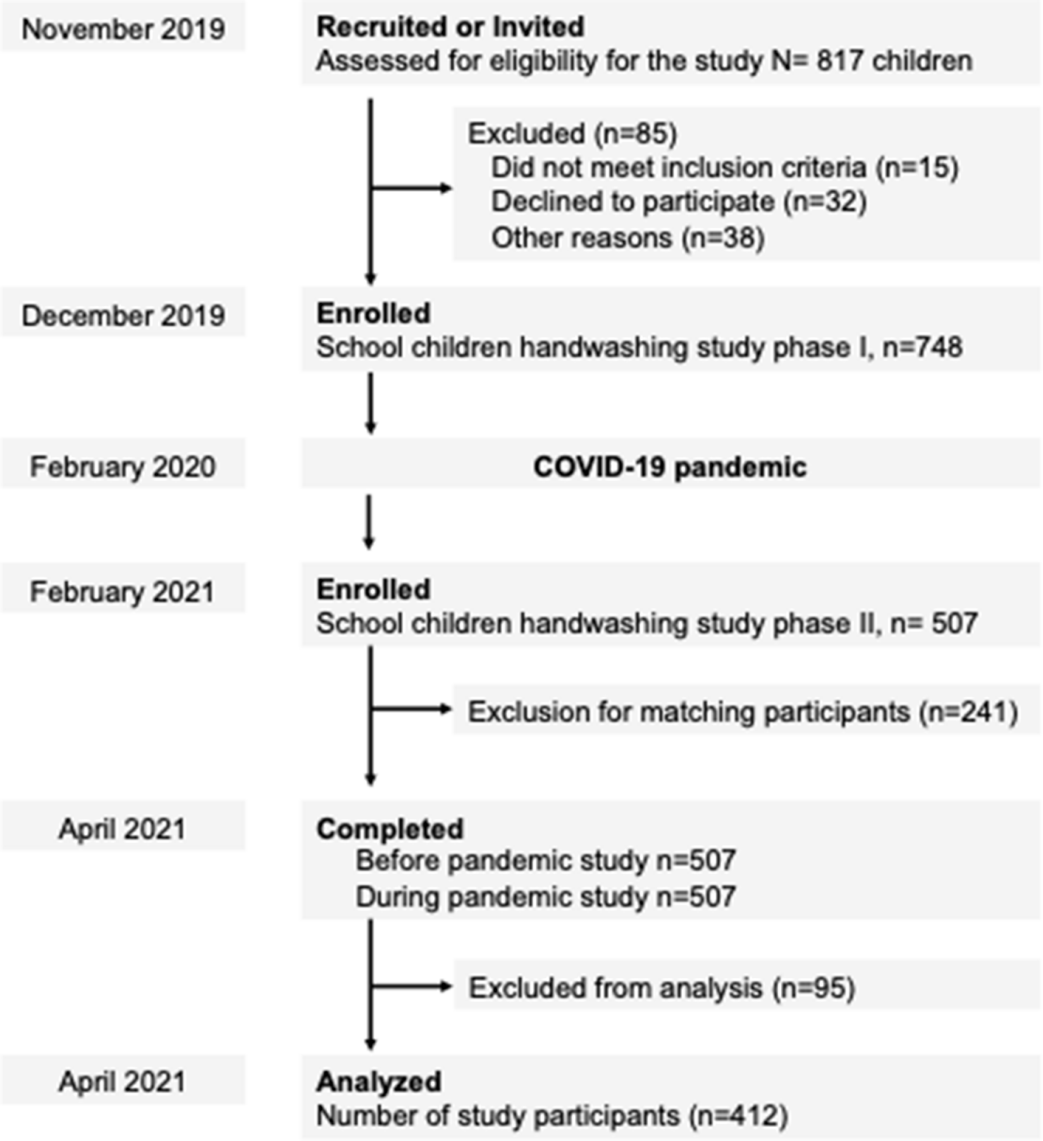
First 817 children assessed for eligible for the study to participate. Before the start of the data collection, an informed consent session was held in a classroom which the study purpose and procedure were explained. Before the pandemic data was collected from a total of 748 participants and during the pandemic was collected a total of 507 participants, respectively. As a result of data cleaning process, a total of 412 participants data were remained which are completely attended both phases.

Eligible participants were students in grades 3–5 who attended the enrolled school, were aged 8–11 years, agreed to participate, and were literate in Mongolia. The researcher provided the school authorities with information about the study to be shared with their parents. The parents and children who agreed to participate provided signed informed consent and assent forms, respectively. A flowchart of participant progression is shown in Figure 1.

### Measures

#### Self-reported questionnaire

The questionnaire used in this study was adapted from previous research on handwashing KAP (14–17). The validity of the questionnaire was assessed using Cronbach’s alpha (*α* = 0.78) and was pretested with elementary schoolchildren from another school in the same district.

The questionnaire consisted of 24 items, divided into three sections:

(1) Handwashing practice: Questions on the frequency of handwashing, use of soap, and handwashing at critical moments (e.g., before eating, and after using the toilet).
(2) Handwashing knowledge: Questions assessing the understanding of disease transmission pathways and proper handwashing techniques.
Attitudes toward handwashing: Questions regarding the perceived importance of handwashing, its role in disease prevention, and awareness of handwashing education.

General sociodemographic data (age, sex, type of accommodation, and parents’ education and employee status) were collected through school records, with parental consent.

### Data collection and analysis

Data were collected at two time points:

(1) Before the pandemic: In December 2019, data were collected in person by the principal investigator and trained researchers using paper-based questionnaires in the classroom. The purpose of the study was explained to the children, and instructions for completing the questionnaires were provided.
(2) During the pandemic: In January 2021, data were collected using the same self-reported questionnaire. The questionnaire was adapted to a Google Forms platform, with each question marked as “required.” Teachers and school administrators were briefed on the study, and parents and children were reminded about their participation via three notices to encourage response rates.

For the analysis, handwashing KAP scores were compared before and during the pandemic:

- Knowledge and attitude: Students received one point for each correct answer on the knowledge and attitude questions. A score of 60% or more on knowledge and attitude items was classified as “sufficient knowledge” and “positive attitude,” respectively.
- Handwashing practice: Handwashing practice was assessed based on four critical moments: (1) before eating, (2) after using the toilet, (3) after visibly touching dirty or unhygienic things, and (4) after coming home. A four-point Likert scale was used, ranging from “always” to “never.” Children who responded “always” for all critical moments were classified as having “proper handwashing” behavior.

The data were entered into Microsoft Excel by two independent researchers. To ensure accuracy, the data entry was cross-checked with the original paper-based records, and discrepancies were corrected.

Descriptive statistics, including means and standard deviations, were used to calculate the data. Statistical analysis was performed using Stata/MP version 14.0 (Stata Corp LP, College Station, TX, USA), and the statistical significance level was set at *p* < 0.05.

Inferential statistics were the dependent variables in the handwashing KAP, whereas the independent variables were sociodemographic factors (e.g., sex and grade, parents’ education, employment status, and accommodation). Odds ratios (ORs) and 95% confidence intervals (CIs) were calculated to assess associations between sociodemographic factors and handwashing behavior. Adjusted odds ratios (AORs) were estimated using multivariate logistic regression models controlling for grade, sex, accommodation type, parents’ education level, and parents’ employment status.

### Ethical considerations

Ethical approval for the study was obtained from the Biomedical Research Ethics Committee of MNUMS (before pandemic data approval number No. 3/09/2019-09-20; during data approval number No. 3/03/2022-03-25). Passive consent was obtained from the parents who were informed that they could decline participation by summiting written refusal. Children gave their assent by raising their hands before the study commenced.

## Results

### Participant characteristics

A total of 399 children completed both phases of data collection: before and during the pandemic COVID-19. The mean age of the children was 9.1 years (SD = 0.1), and 40.8% of the children were male (Table 1). The majority of children lived in apartments (68.8%).

**Table 1:** Characteristics of participants (*n* = 339).

Variables	Frequency	Percent
Age (Mean, SD)	9.1	0.0
Gender		
Boys	163	40.8
Girls	236	59.2
Grade		
Third	120	30.1
Fourth	105	26.3
Fifth	174	43.6
Accommodations		
Ger*	34	8.6
Simple house	89	22.6
Apartment	271	68.8

*Traditional Mongolian dwelling.

### Changes in handwashing knowledge

Handwashing knowledge was assessed using 14 questions, and the results were presented as the average score and percentage of correct responses. Before and during the COVID-19 pandemic, knowledge was assessed, with the average handwashing knowledge score being 9.8 (SD = 2.5, range: 1–14) before the pandemic and 12.3 (SD = 1.6, range: 1–14) during the pandemic (Table 2). The percentage of correct responses regarding critical handwashing times increased significantly, from 23.0% before the pandemic to 80.4% during the pandemic and from 83.7 to 99.5% for the importance of handwashing at critical times. A total of 128 (32.1%) children scored having sufficient knowledge before the pandemic and 296 (71.2%) during the pandemic. The number of children who answered all attitude questions correctly was 14.3% (*n* = 59) before the pandemic, and it slightly increased to 26% (*n* = 109) during the pandemic. Both boys and girls showed a significant increase in handwashing knowledge, with the knowledge score for boys increasing from 9.2 to 12.0 (*p* < 0.001) and for girls from 10.3 to 12.4 (*p* < 0.001). Significant improvement was observed across all grades, with third graders showing an increase from 9.1 to 12.3 (*p* < 0.001), fourth graders from 10.7 to 12.6 (*p* < 0.001), and fifth graders from 9.9 to 12.1 (*p* < 0.001). Children with fathers having secondary education increased from 9.5 to 12.2 (*p* < 0.001), those with college-educated fathers from 9.5 to 12.3 (*p* < 0.001), and those with fathers having above-college education from 10.2 to 12.3 (*p* < 0.001). A similar trend was observed in mothers’ education.

**Table 2:** Average score of children’s HW-related knowledge and attitude.

Variables	Knowledge score (range: 1–14)		*p*-value	Attitude score (range: 1–16)		*p*-value
	Before COVID-19	During COVID-19		Before COVID-19	During COVID-19	
Overall	9.8 ± 2.5	12.3 ± 1.6	0.00	8.2 ± 1.3	8.7 ± 1.0	0.00
Gender						
Boys (*n* = 163)	9.2 ± 2.7	12.0 ± 1.7	0.00	7.9 ± 1.5	8.6 ± 1.1	0.00
Girls (*n* = 236)	10.3 ± 2.3	12.4 ± 1.5	0.00	8.3 ± 1.3	8.7 ± 1.0	0.00
Grade						
3rd grade (*n* = 120)	9.1 ± 2.5	12.3 ± 1.6	0.00	7.6 ± 1.4	8.6 ± 1.1	0.00
4th grade (*n* = 105)	10.7 ± 2.3	12.6 ± 1.5	0.00	8.5 ± 1.2	8.8 ± 1.1	0.09
5th grade (*n* = 174)	9.9 ± 2.5	12.1 ± 1.6	0.00	8.3 ± 1.3	8.6 ± 1.1	0.00
Accommodation						
Ger (*n* = 34)	9.8 ± 2.9	12.3 ± 1.4	0.00	8.0 ± 1.3	8.8 ± 1.1	0.00
Simple house (*n* = 89)	9.7 ± 2.6	12.2 ± 1.7	0.00	7.8 ± 1.5	8.7 ± 1.0	0.00
Apartment (*n* = 271)	9.9 ± 2.5	12.3 ± 1.6	0.00	8.3 ± 1.3	8.7 ± 1.1	0.00
Father education						
Secondary	9.5 ± 2.7	12.2 ± 1.6	0.00	8.0 ± 1.3	8.7 ± 1.0	0.00
College	9.5 ± 2.5	12.3 ± 1.5	0.00	7.8 ± 1.3	8.7 ± 0.9	0.00
Above college	10.2 ± 2.3	12.3 ± 1.5	0.00	8.3 ± 1.3	8.6 ± 1.1	0.00
Mother education						
Secondary	9.2 ± 2.6	12.2 ± 1.6	0.00	8.0 ± 1.4	8.8 ± 0.9	0.00
College	10.3 ± 2.3	12.7 ± 1.4	0.00	8.1 ± 1.4	9.7 ± 0.9	0.00
Above college	9.9 ± 2.5	12.2 ± 1.6	0.00	8.2 ± 1.4	8.6 ± 1.1	0.00

The effect size for the change in handwashing knowledge was calculated using Cohen’s *d*, which yielded a moderate effect size of [*d*-value = 0.69], indicating meaningful improvement in handwashing knowledge during the pandemic. The significant increase in handwashing knowledge during the pandemic may reflect heightened awareness and education regarding hygiene practices, particularly in response to the COVID-19 pandemic. Overall, there was a marked improvement in both handwashing knowledge and attitudes among participants before and during the pandemic. The most notable increases were observed in children’s knowledge regarding whether human feces contain germs, as well as in their attitudes about the critical moments for washing hands, including after coughing, blowing their nose, and before eating.

### Changes in handwashing attitudes

Attitudes toward handwashing were assessed using 10 items, and the results were expressed as the average score and percentage of correct responses. Before and during the COVID-19 pandemic, children’s attitudes were assessed, with an average handwashing attitude score of 8.2 (SD *=* 1.3, range: 1–16) before the pandemic and 8.7 (SD *=* 1.0, range: 1–16) during the pandemic (Table 2).

A total of 332 (80%) children had positive attitudes before and during the pandemic. A significant improvement in attitudes was also observed for both boys (from 7.9 to 8.6, *p* < 0.001) and girls (8.3–8.7, *p* < 0.001), demonstrating that the pandemic had a generally positive effect on attitudes toward handwashing for all children. Improvements in attitudes toward handwashing were observed across all groups, with children in a ger increasing from 8.0 to 8.8 (*p* < 0.001), those in a simple house from 7.8 to 8.7 (*p* < 0.001), and those in an apartment from 8.3 to 8.7 (*p* < 0.001). Children of parents with secondary education showed an increase from 8.0 to 8.8 (*p* < 0.001), whereas children with college-educated parents increased from 8.1 to 9.7 (*p* < 0.001), and those with above-college-educated parents showed a more modest increase from 8.2 to 8.6 (*p* < 0.001).

### Changes in handwashing practice

Handwashing-related practice was assessed using six questions, and the results are expressed as the percentage of correct responses (Table 3).

**Table 3:** Schoolchildren’s handwashing practice responses rate (*n* = 399).

Variables	Before pandemic		During pandemic		*p*-value^*^
	N	%	N	%	
How did you wash your hands?					
Just water	98	31.8	26	6.3	0.000
Soap and water	151	33.7	356	89.6	
Use wet tissue	52	11.9	3	0.7	
Use hand sanitizer	29	6.8	10	2.4	
Never wash	69	15.8	4	1.0	
How many times did you wash your hands?					
Less than 2 times	75	18.8	57	14.3	0.000
3–5 times	261	65.4	272	68.2	
More than 6 times	63	15.8	70	17.5	

*Pearson chi-square.

**Figure 2:**
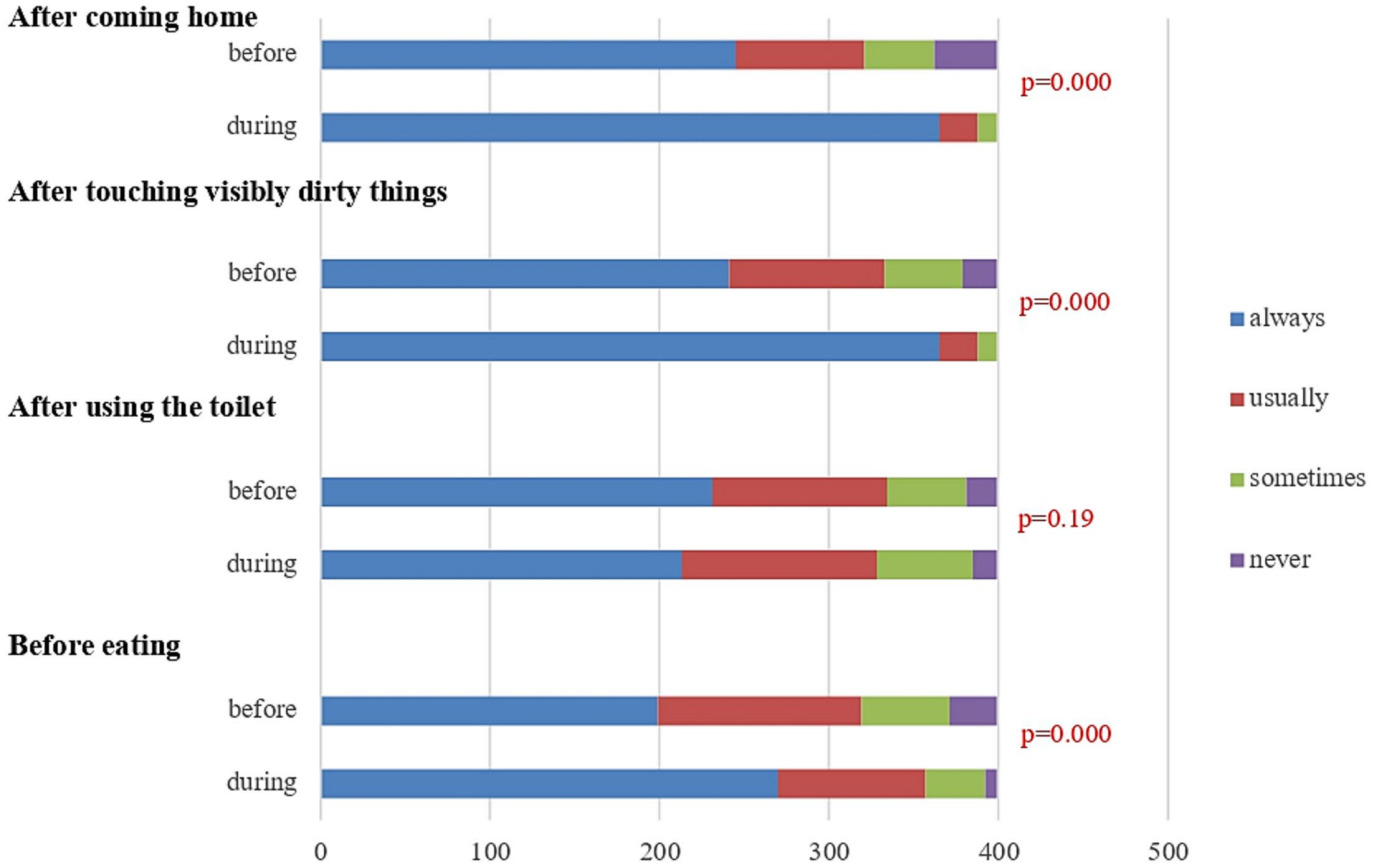
Handwashing practices: 4 critical moments before and during the pandemic. Bar graph illustrates self-reported handwashing behavior at four key moments: after coming home, after touching visibly dirty things, after using the toilet, and before eating. Responses are categorized into four frequencies: “always,” “usually,” “sometimes,” and “never.” This figure illustrates self-reported handwashing behaviors at four key moments: after coming home, after touching visibly dirty things, after using the toilet, and before eating. Comparisons between behaviors before and during the pandemic are shown for each moment, Statistically significant improvements in handwashing frequency during the pandemic are observed in three of the four situations (*p* < 0.05), except for after using the toilet (*p* = 0.19).

Handwashing practice was assessed based on four critical moments: (1) after using the toilet, (2) before eating meals, (3) after touching visibly dirty or unhygienic objects, and (4) after coming home (Figure 2). These moments were selected based on the children’s critical handwashing moments, and each of these moments was scored using a four-point Likert scale, ranging from “always” to “never.” Children who reported “always” for all four criteria were classified as having “proper handwashing practice.” Handwashing at critical moments showed a significant increase, apart from “before eating,” in general. The lack of change in handwashing before eating may reflect reduced social cues during the COVID-19 pandemic, as distancing measures limit group meals and adult supervision, potentially weakening routine hand hygiene behavior. Handwashing frequency increased, possibly influenced by COVID-19 interventions, such as the widespread transmission of health information.

### Sociodemographic factors and handwashing behavior

Girls were significantly more likely to engage in proper handwashing than boys. The crude odds ratio (COR) for girls was 2.96 (95% CI = 1.71–5.14, *p* < 0.01), and the AOR was 4.50 (95% CI = 2.22–9.12, *p* < 0.01), indicating over four times higher odds for girls. The grade level also influenced handwashing behavior. Fourth-grade students had higher odds of proper handwashing compared to third-grade students (COR = 4.46, 95% CI = 1.86–10.68, *p* < 0.01; AOR = 5.27, 95% CI = 1.75–15.87, *p* < 0.01). The association for the fifth-grade students was not significant. Father’s education was positively associated with proper handwashing. The COR for fathers with education above college was 2.12 (95% CI = 1.16–3.87, *p* = 0.02), and the AOR was 2.63 (95% CI = 1.07–6.45, *p* = 0.03). Maternal self-employment was significantly linked to better handwashing behavior. The COR for self-employed mothers was 1.88 (95% CI = 0.83–4.24), and the AOR was 2.71 (95% CI = 1.01–7.25, *p* = 0.04). Father’s employment in public service was negatively associated (COR = 0.51, 95% CI = 0.22–1.20; AOR = 0.35, 95% CI = 0.12–0.98, *p* = 0.04) with proper handwashing (Table 4).

**Table 4:** Logistic regression results for factors influencing proper handwashing behavior.

Explanatory variables		Handwashing behavior	
		COR	AOR
Gender	Boys	1.00	1.00
	Girls	2.96 [1.71–5.14]**	4.50 [2.22–9.12]**
Grade	Third grade	1.00	1.00
	Fourth grade	4.46 [1.86–10.68]**	5.27 [1.75–15.87]**
	Fifth grade	1.59 [0.89–2.83]	1.92 [0.89–4.16]
Accommodation	Ger	1.00	1.00
	Simple house	0.89 [0.33–2.35]	0.68 [0.19–2.38]
	Apartment	1.59 [0.64–3.90]	0.89 [0.28–2.79]
Maternal education	Secondary	1.00	1.00
	College	2.00 [0.68–5.81]	1.29 [0.36–4.54]
	Above college	1.54 [0.75–3.16]	0.99 [0.35–2.77]
Father’s education	Secondary	1.00	1.00
	College	1.17 [0.50–2.73]	0.89 [0.30–2.64]
	Above college	2.12 [1.16–3.87]*	2.63 [1.07–6.45]*
Maternal employee	Private company	1.00	1.00
	Self-employee	1.88 [0.83–4.24]	2.71 [1.01–7.25]*
	Public service	1.01 [0.48–2.10]	1.16 [0.46–2.92]
	Unemployed	2.04 [0.88–4.71]	2.45 [0.97–6.15]
Father’s employment	Private company	1.00	1.00
	Self-employee	0.54 [0.27–1.07]	0.43 [0.19–1.00]
	Public service	0.51 [0.22–1.20]	0.35 [0.12–0.98]*
	Unemployed	1.05 [0.22–5.03]	0.55 [0.94–3.23]

*p < 0.05; **p < 0.01.

Overall handwashing KAP scores increased significantly during the pandemic compared to before (mean difference = 3.68, 95% CI = [4.06, 3.29]). The paired *t*-test yielded a *p*-value < 0.0000, providing strong evidence against the null hypothesis (which assumes no difference). Therefore, we can confidently reject the null hypothesis and conclude that there was a significant improvement in handwashing behavior during the pandemic.

## Discussion

Schoolchildren play a significant role in the spread of respiratory infections due to the close interactions between schools and their communities (18, 19). Many countries are concerned about the preparedness of handwashing facilities and school children’s KAP.

In this study from February 2021 (as shown in the previous section), more schoolchildren remembered washing their hands after using the toilet, after touching visibly dirty things, and after coming home compared to responses in December 2019.

The observed increase in handwashing behaviors, particularly after key moments such as using the toilet or touching dirty objects, is likely linked to heightened public health messaging and media interventions urging safety and hygiene practices during the lockdown. During the lockdown, the population was restricted to social gatherings and restaurant visits. It is possible that eating at home during the lockdown led to a decrease in the frequency of handwashing before meals.

Our study showed an increase during the pandemic, a pattern observed in other contexts. For example, Haston et al. found that the percentage of U. S. adults who reported remembering to wash their hands in certain circumstances increased during the pandemic compared to before the pandemic. Studies conducted on schoolchildren during the previous influenza pandemic (20, 21) and the COVID-19 pandemic (22) have shown similar outcomes to our results.

While several studies have examined hand hygiene behaviors among schoolchildren, they have primarily focused on either the pre-pandemic or during-pandemic period in isolation (5, 23, 24). Comparative studies assessing changes across both periods remain limited. This study addresses this gap by providing a direct comparison across two time points, thereby contributing valuable longitudinal insights into the impact of the pandemic on hygiene-related behavior among children. Previous studies in Mongolia found that only 50.1% of schoolchildren practiced handwashing at critical times. Poor hygiene was linked to the male sex, larger households, and a lack of school facilities and soap (16).

Washing hands with soap and water is the most effective method for preventing communicable diseases, particularly among children. However, when soap and water are not available, the Centers for Disease Control and Prevention (CDC) recommends using alcohol-based hand sanitizers that contain at least 60% alcohol (25). According to the results, it appears more children use wet tissues (*n* = 52, 11.9%) than alcohol-based sanitizers (*n* = 29, 6.9%) which means that children’s behavior is very different from that of university students. University students are known to differ in their daily disinfections (26). Unfortunately, baby wipes or wet tissues are not recommended for effectively removing germs in all settings, as they are not designed to kill pathogens.

### Handwashing knowledge and attitude

In Mongolia, handwashing education is a knowledge-based teaching approach, and most schools have a crowded environment, especially elementary schools. Since 2002, teachers have offered official handwashing education classes to only fourth-grade students for 40 min. The class covers a brief introduction to germs, hand hygiene, and handwashing instructions (27). Apart from this, there is no specific instruction for the teachers. Generally, there are no routine, repetitive instructions or school policies on hand hygiene. An analysis of the 2014 Situation of Children in Mongolia report found that the majority of public schools were overcrowded and had limited access to proper sanitation facilities. The promotion and delivery of health education for children were also insufficient due to a shortage of resources, including teaching aids (28). Educational interventions that use visual tools to teach proper handwashing techniques to students in primary schools have improved children’s knowledge and awareness of appropriate hand hygiene and increased adherence to handwashing (29, 30). In a study by Grover et al. (23), the findings were encouraging; high-intensity and interactive handwashing interventions could be more effective than traditional knowledge-based classes. They believed that traditional knowledge-based teaching and teaching hours were insufficient to explain the low frequency of handwashing practice. Consequently, the quality and frequency of health programs should be evaluated and updated to meet the needs of schoolchildren of various ages, and non-traditional framework experiences should be learned.

Our findings align with the behavior change theories proposed by Lau et al. (31), which emphasize the importance of both knowledge and social factors in shaping health behaviors. Despite limited formal education on the topic, the increase in children’s handwashing knowledge suggests that information dissemination through media and family could have been a significant contributing factor (32–35). Health behavior awareness is a critical factor in behavior change theory, and a successful intervention may need to address other factors, such as social support and personal competencies (36). In this study, knowledge, the overall score, and those who answered all knowledge questions correctly increased the number of participants before the pandemic. According to a report by Sian (37), handwashing education prior to the pandemic was not emphasized, and simply providing disease-related information was not sufficient to change behavior.

Our findings align with those of a large-scale study conducted on South Korean adolescents, which reported a significant improvement in hand hygiene behaviors during the early stages of the COVID-19 pandemic (38). This increase was largely attributed to extensive public health campaigns, school closures, and heightened awareness of the importance of hand hygiene in preventing the spread of the virus. Similarly, in our study, we observed a marked improvement in children’s knowledge of and attitudes toward hand hygiene during the pandemic, reflecting the impact of heightened public health messaging and pandemic-driven interventions.

### Schoolchildren’s handwashing practice

Previous studies have suggested that action-oriented, participatory approaches to health and hygiene education interventions, such as those that engage pupils in developing ideas about behavioral changes and actions to carry them out, may be equally or more effective than didactic, conventional education. In addition, visually stimulating and participatory teaching methods are strongly associated with effective handwashing techniques among school children (39). Sex-based differences in hygiene behavior observed in this study, particularly the lower engagement of boys in proper handwashing practices, highlight the need for targeted interventions in schools. Active teaching and learning approaches may encourage pupils’ full participation and ownership of the behavior change, resulting in increased knowledge and more sustained or effective adoption of the behavior (40). Handwashing practices may require constant reinforcement from the adults around them (teachers and parents). However, COVID-19-related health education or prevention/promotion messages target the community, not children in particular. The following factors may have contributed to the message and health promotion reaching children without failure: frequent newness, fear of the virus, emergency situations, and home lockdown. Children imitate their parents’ behavior, which influences their handwashing practices.

While the study design captures meaningful changes in children’s handwashing behavior, it is important to acknowledge the potential biases associated with self-reported data and the limitations of comparing behaviors during a pandemic when children’s daily routines are disrupted. Future research could explore the sustainability of these improved hygiene behaviors beyond the pandemic context, examining whether these habits persist once normal school routines are fully restored. Additionally, exploring the role of parental involvement and school-based interventions in shaping children’s hygiene practices could provide deeper insights into effective behavior change strategies. Research among schoolchildren in low-and middle-income countries consistently shows low rates of handwashing after toilet use, typically ranging between 7 and 15%. Despite the relevance of public health, few studies have specifically focused on assessing hand hygiene practices among elementary school-aged children (41, 42).

In kindergarten, children typically learn through observation and imitation. However, in schools, the learning environment changes—lessons, including health education, are usually taught by a single teacher, which may limit opportunities for specialized or interactive hygiene instruction. This highlights a key difference in the learning environments between kindergarten and school which should be considered when designing hygiene education programs. Based on the results of this study and previous evidence, we recommend the implementation of interactive and participatory handwashing classes in elementary schools in Mongolia.

## Conclusion

This study demonstrated a significant improvement in handwashing KAP among primary schoolchildren in Ulaanbaatar, Mongolia, during the COVID-19 pandemic. The results indicate that the pandemic, with its widespread public health messaging and disruption of normal routines, played a critical role in shaping children’s hygiene behaviors. Specifically, children showed notable increases in their knowledge of proper handwashing techniques, importance of hand hygiene at critical moments, and their attitudes toward hygiene practices. The study also reveals that while knowledge and attitudes have improved, actual handwashing practices have varied, especially regarding behavior before eating meals. This suggests that while education and awareness are essential, sustained behavioral change may require ongoing reinforcement and targeted interventions, particularly within school environments. It is crucial for public health policies to prioritize hand hygiene education, not only during pandemics but also as part of routine school health curricula, to ensure that students have consistent, age-appropriate learning opportunities. In conclusion, the COVID-19 pandemic has provided an unprecedented opportunity to understand and improve children’s health behaviors, especially regarding hand hygiene. This study contributes valuable insights into how global health crises can shape public health practices in vulnerable populations and calls for continued investment in educational initiatives that promote health literacy and sustainable behavioral change.

## Data Availability

The raw data supporting the conclusions of this article will be made available by the authors, without undue reservation.
